# Reliability of the Japanese Version of the Kit for Assessment of Time Processing Ability (KaTid)-Child in Typically Developing Children: A Pilot Study

**DOI:** 10.7759/cureus.99921

**Published:** 2025-12-23

**Authors:** Shota Tasaka, Yuko Nishiura, Misako Sano, Toyohiro Hamaguchi

**Affiliations:** 1 Graduate School of Health Sciences, Department of Rehabilitation, Saitama Prefectural University, Koshigaya, JPN; 2 Graduate School of Medicine, Department of Occupational Therapy, Nagoya University, Nagoya, JPN

**Keywords:** children, inter-rater reliability, katid-child, occupational therapy, reliability, test–retest, time processing ability

## Abstract

Background: Children with developmental disabilities often experience difficulties in time management, which negatively affect their daily functioning and social adjustment. The Kit for Assessment of Time Processing Ability (KaTid) is a validated Swedish instrument for assessing time management skills. However, its use in Japan remains limited, and a culturally adapted version has not yet been validated.

Objective: This study aimed to validate the Japanese version of the KaTid-Child (KaTid-C-J) by examining its test-retest reliability, inter-rater reliability, and comparability of Time Processing Ability (TPA) scores with the developmental age bands of the original Swedish KaTid-Child.

Methods: Twelve typically developing children aged 5-10 years were recruited through snowball sampling. The KaTid-C-J was administered twice by the same examiner with an interval of 30-60 minutes to assess test-retest reliability. Video recordings were independently scored by another rater to assess inter-rater reliability. Spearman’s correlation, Bland-Altman plots, Cohen’s kappa, and intraclass correlation coefficients (ICCs) were used to examine reliability. Bootstrapping was performed to estimate 95% confidence intervals (CIs). Comparisons between the Japanese and Swedish versions were made using chi-squared tests.

Results: Test-retest reliability showed moderate-to-strong correlations (Spearman’s ρ = 0.531-0.872) across four domains of time processing (TP, TO/TC, TO/OT, TM), with no systematic or random errors. Inter-rater reliability was perfect, with both kappa and ICC values of 1.00. The Japanese and Swedish versions demonstrated high concordance for children aged 8-10 years; however, children aged 5-7 years showed slightly higher ability scores in the Japanese version, possibly due to linguistic and cultural differences in month-naming conventions.

Conclusions: The Japanese version of the KaTid-Child demonstrated high test-retest and inter-rater reliability, supporting its potential as a reliable tool for assessing time processing ability in typically developing Japanese children. Further studies with larger and more diverse samples are needed to confirm its validity and cross-cultural applicability.

## Introduction

Autism spectrum disorder (ASD) is a neurodevelopmental disorder characterized by persistent deficits in social communication and restricted, repetitive patterns of behavior. The Diagnostic and Statistical Manual of Mental Disorders, Fifth Edition (DSM-5), classifies ASD alongside other neurodevelopmental disorders such as attention-deficit/hyperactivity disorder (ADHD) [1]. According to the most recent surveillance data from the Centers for Disease Control and Prevention (CDC), approximately one in 36 children aged eight years in the United States was diagnosed with ASD in 2020 [2]. This prevalence represents a continued increase from previous estimates - one in 54 in 2016 [3]. ASD has been found to affect children across all racial, ethnic, and socioeconomic groups, with a prevalence approximately four times higher among boys than girls [2]. These trends underscore the growing importance of early identification and intervention for children with ASD.

The World Health Organization (WHO) defines neurodevelopmental disorders in the International Classification of Diseases 11th Revision as a group of conditions that appear in early childhood or during development and that affect cognitive, motor, behavioral, social, and language functions. These include intellectual disability, ASD, ADHD, developmental coordination disorder (DCD), tic disorders, and pervasive developmental disorders [4].

In Japan, developmental disabilities are defined as “Autism, Asperger’s syndrome and other pervasive developmental disorders, learning disabilities, attention-deficit/hyperactivity disorder and other similar disorders of brain functions whose symptoms usually manifest at a young age” (as stated in the 2004 Act on Support for Persons with Developmental Disabilities) [5]. According to the Japanese Ministry of Education, Culture, Sports, Science and Technology [6], approximately 6.5% of children in mainstream elementary and junior high school classes exhibit characteristics of developmental disabilities. A population-based study in Hirosaki City reported an adjusted prevalence of ASD among five-year-old children to be at 3.22% (95% confidence interval (CI): 2.66-3.76%), with a male-to-female ratio of 2.2:1 [7].

Caregivers and advocates of children with developmental disabilities often report various challenges they face, such as the children’s “inability to adapt to the environment” and “struggling with shifting between tasks or activities” [8]. Effective time management is critical in helping such children navigate their daily lives. The WHO’s International Classification of Functioning, Disability and Health for Children and Youth (ICF-CY) defines time management as a higher-level cognitive function, describing it as “mental functions of ordering events in chronological sequence, allocating amounts of time to events and activities” [9].

Recent research has further emphasized that interventions targeting time processing ability can significantly improve everyday functioning in children, highlighting the necessity of accurate assessment and support [10].

Children who "cannot get ready in the morning" despite having no motor impairments may experience difficulty ascertaining the time required to start and complete tasks. Time management and related skills, such as time awareness and action planning, are essential for functioning in social settings, such as in school and at work. According to the DSM-5 [1], difficulties in both “adapting behavior to different social situations” and “engaging in imaginative play or making friends” are indicators of developmental disabilities. Adapting one’s behavior to social situations requires higher-order cognitive functions. Delays in the development of time management skills may lead to a lack of time awareness; this can make it difficult to gauge when to begin actions to complete planned tasks within a stipulated time. This can negatively impact punctuality and coordination with others [11-14].

The Kit for assessment of Time processing ability (KaTid), developed by Janeslätt et al. [11] in Sweden, assesses the development of time management and provides treatment and support. Time processing ability (TPA) in the KaTid includes time perception (TP), time orientation (TO), and time management (TM). The ICF-CY defines these as the experience of time (b1802), orientation to time (b1140), and time management (b1642), respectively [9,12].

The KaTid has been effective in distinguishing between the time management skills of children with typical development and those with developmental disabilities. It assesses TP, time orientation/time concept (TO/TC), time orientation/objective time (TO/OT), and TM, which helps identify areas that require developmental support. Previous studies using the KaTid demonstrated its efficacy in differentiating between children with and without developmental disabilities based on Rasch analysis and practical assessment of both typically developing children and those with developmental disabilities [11-14].

Although the KaTid has been used to examine Swedish children, no study outside Sweden has utilized this scale. In Japan, support for time management difficulties has been provided through some tools, such as the quarter-hour watch and timers [15,16]. However, a developmental assessment index, such as the KaTid, is lacking in Japan. A Japanese version of the KaTid-Child that can demonstrate test-retest and inter-rater reliability to assess time management skills and effectively support development is essential.

To use the Japanese version of the KaTid-Child, validating the cultural relevance of the questions and correlating them with standardized tests in Japan is necessary. This study aimed to verify the test-retest reliability and inter-rater reliability and compare Japanese children’s TPA scores with the developmental age bands of the original KaTid-Child.

## Materials and methods

Study design

The KaTid-C-J was used to evaluate time processing ability (TPA) in Japanese children.

Instrument

The KaTid-Child assesses children’s development of the concept of time. The Japanese version consists of four domains: (A) time perception (TP), (B) time orientation/time concept (TO/TC), (C) time orientation/objective time (TO/OT), and (D) time management (TM) [12].

Translation

The KaTid-C-J was translated from English to Japanese by the first author, with permission from the original author, and subsequently back-translated by another researcher. The back-translated version was reviewed and approved by the original author.

KaTid testers

To properly conduct the KaTid-C-J assessments, one of the two raters completed an online course on TPA directly from the first author. The other scorer, who did not take the course, received an explanation of the scoring method from the trained rater based on the Japanese version of the manual.

Procedure

Participant Recruitment

Through the researcher’s network, in July 2021, 15 “Request for your cooperation for participating in the study” emails were sent to families with children living in and near Saitama Prefecture, Japan. A snowball sampling method within local community networks was used, and respondents were asked to introduce the researcher to other potential participants. A total of 17 responses were received between July and December 2021, which constituted the data collection period. Participants’ ages were confirmed, and assessment schedules were arranged via email. Participation was voluntary. On the day of assessment, the study purpose and measures for the protection of personal information were verbally explained, and written informed consent was obtained. Assessments were rescheduled if a respondent cancelled owing to illness.

Eligibility criteria included children aged 5-10 years with no diagnosis of autism spectrum disorder (ASD), attention-deficit/hyperactivity disorder (ADHD), or developmental coordination disorder (DCD). Exclusion criteria included children with a confirmed diagnosis of ASD, ADHD, or DCD; children for whom assessment scheduling could not be arranged; children who cancelled participation due to illness and could not be rescheduled; and children who were unable to complete the assessment protocol. Although no participants were excluded based on age, five respondents withdrew prior to assessment. Ultimately, 12 children participated in this pilot study.

A post-hoc power analysis confirmed that this sample size provided sufficient statistical power (>0.80) to detect strong correlations (r > 0.80), which was the primary aim of the present reliability analysis.

Assessment Environment

The administration of KaTid-C-J was conducted in a quiet room with only family members present. The participant sat on the floor or in a chair, with a desk in front of them. A video recording was created for scoring purposes.

Assessment of Test-Retest Reliability

The same investigator, an occupational therapist, administered the assessment twice to the same child. Each session lasted approximately 30 minutes, separated by an interval of 30 minutes to one hour. This short interval was adopted to accommodate practical constraints during the COVID-19 pandemic and limited access to participants. The child’s sense of time was assessed without feedback to avoid influencing the developmental process.

Assessment of Inter-Rater Reliability

The rater independently assessed the same child on two separate occasions using the KaTid-C-J. The tests were video-recorded, and the recordings were subsequently reviewed and scored separately by a rater and a scorer. This approach ensured that both individuals independently evaluated the child’s performance, thereby avoiding the potential physical and mental fatigue that can arise from repeated testing.

Comparison of the KaTid-Child Versions

Data from the intra-test-retest reliability assessments were used to calculate developmental ages (scale points) via logits from the original study data to the children’s total raw scores [11].

Statistical analysis

Distribution and Assessment Error

The distribution of the KaTid-C-J scores was confirmed via Spearman’s rank correlation and probability density curves. Assessment errors were evaluated via Bland-Altman plots to check for random and systematic errors.

Verification of Test-Retest Reliability

Results of the two repeated assessments were used to score domains A-D, and the correlation coefficients were calculated. The 95% confidence intervals (CIs) were calculated via block bootstrapping. Kappa values and intraclass correlation coefficients (ICCs) were determined according to Landis and Koch’s criteria [17].

Verification of Inter-Rater Reliability

Scores from one examiner’s assessment and another scorer’s video-recorded assessment were used to calculate kappa values and ICCs for domains A-D. Data were resampled 1,000 times via block bootstrapping to calculate the 95% CIs.

Comparison of the Japanese and Swedish Versions

The total scores of the two assessments were converted into scale scores via logits from the original study’s data. Agreement between the Japanese and Swedish versions was assessed through a chi-squared (χ2) test with stratified analysis by age group.

Ethical considerations

This study was approved by the Ethics Committee of Saitama Prefectural University (No. 21505). Written informed consent was obtained from the parents or legal guardians. In addition to parental consent, written informed assent was obtained from all participating children (aged 5-10 years) following age-appropriate explanation using a child-friendly information sheet. Data handling procedures ensured the protection of sensitive personal information, with a designated person responsible for data management.

## Results

Participants

**Figure 1:**
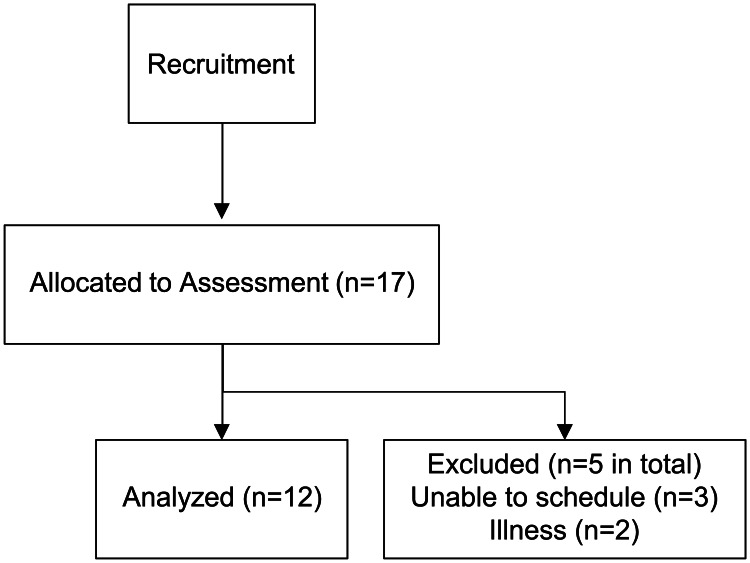
Study flow diagram This flowchart illustrates the participant recruitment and allocation processes. Of the 17 recruited participants, 12 were analyzed after five were excluded owing to scheduling conflicts (n = 3) or illness (n = 2).

In total, 12 participants were included in this study. Figure 1 outlines the recruitment process of participating children. Table 1 presents their age and sex. All scheduled participants agreed to the verbal explanation and provided written informed consent.

**Table 1:** Participating children’s age and sex Note: This table presents the age and sex distribution of the 12 participating children. The sample comprises 10 males and two females, ages ranging from five years 11 months to 10 years 11 months.

Case	Age (in years and months)	Sex
1	5y 11m	Male
2	6y 4m	Male
3	7y 6m	Male
4	8y 10m	Male
5	9y 4m	Male
6	9y 5m	Female
7	10y 0m	Male
8	10y 2m	Male
9	10y 3m	Male
10	10y 9m	Female
11	10y 10m	Male
12	10y 11m	Male

Test-retest reliability

**Figure 2:**
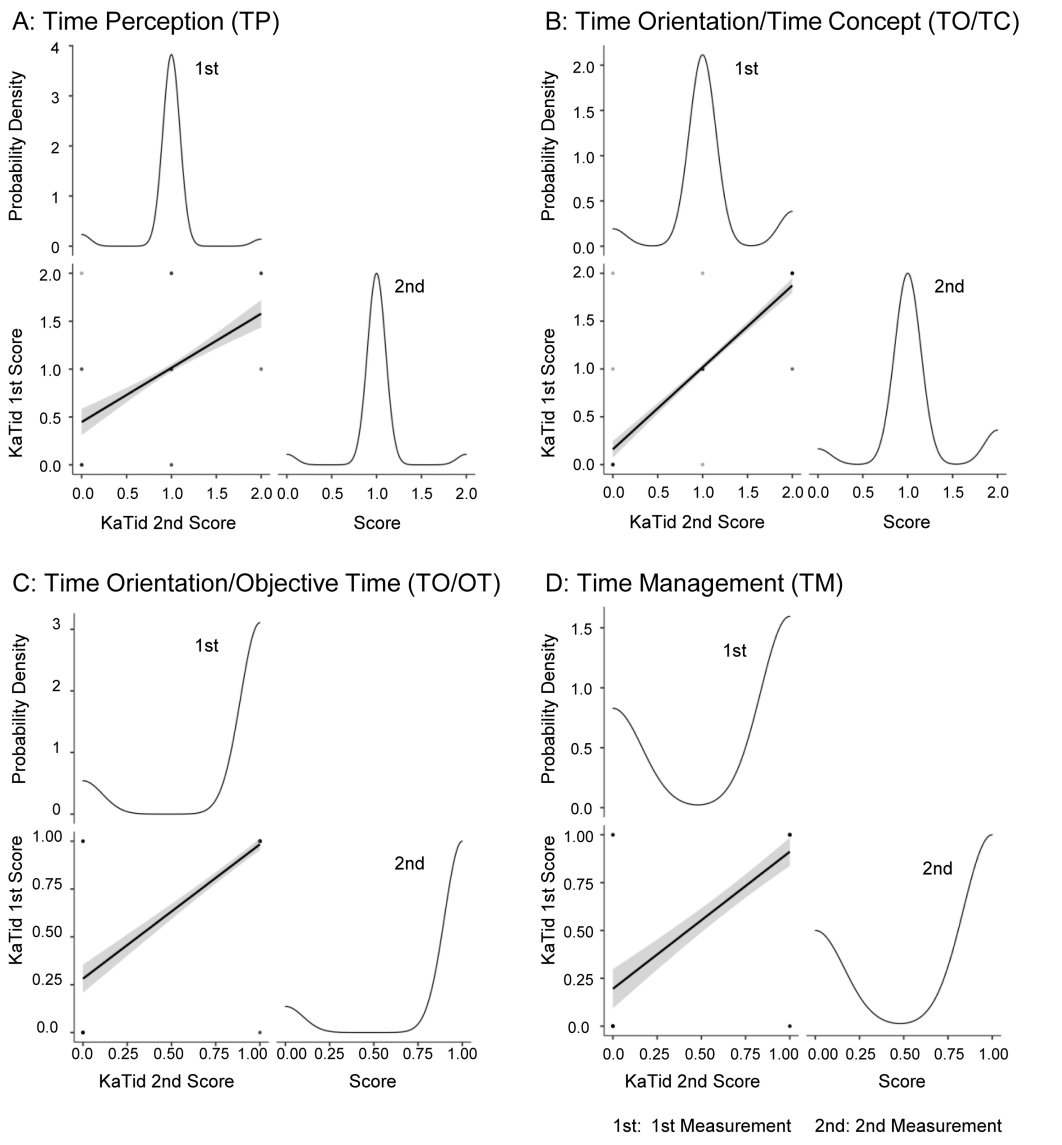
Test-retest reliability scatterplots and probability density curves These figures illustrate the test-retest reliability and probability density of the scores (0, 1, 2) for the initial and subsequent assessments across the four domains: TP, TO/TC, TO/OT, and TM. Spearman’s rank correlation is illustrated in the scatterplots. Probability density curves represent the score distributions for both assessments.

To assess retest reliability, data from the 12 participants, the number of items (A = 15, B = 14, C = 18, D = 10), and two assessments were used. Bland-Altman plots confirmed the absence of random and systematic errors; random errors for the data from the two assessments were A: TP (t = −1, df = 179, p = 0.3), B: TO/TC (t = −0.33, df = 167, p = 0.7), C: TO/OT (t = −1.7, df = 215, p = 0.08), and D: TM (t = −0.26, df = 119, p = 0.8). Systematic errors were A: TP (n = 180, bias = −0.022, 95% CI = −0.066 to 0.022), B: TO/TC (n = 168, bias = 0.006, 95% CI = −0.041 to 0.029), C: TO/OT (n = 216, bias = 0.028, 95% CI = −0.059 to 0.004), and D: TM (n = 180, bias = −0.008, 95% CI = −0.073 to 0.056). The Bland-Altman plots showed that the mean differences between the two assessments were close to zero and that most data points were within the 95% limits of agreement, indicating no systematic bias and consistent scoring across the score range. Spearman’s rank correlation coefficients (ρ) were 0.531, 0.872, 0.767, and 0.721, respectively. Figure 2 illustrates the scatter plots and probability density curves for the first and second assessments. Table 2 presents the results of Spearman’s rank correlation, kappa values, ICCs, and bootstrapping for within-rater reliability.

**Table 2:** Test-retest reliability verification Note: This table summarizes the test–retest reliability of the KaTid-Child Japanese version (KaTid-C-J) across four domains: time perception (TP), time orientation/time concept (TO/TC), time orientation/objective time (TO/OT), and time management (TM). Spearman’s rank correlation coefficients (ρ), kappa values, intraclass correlation coefficients (ICCs), and 95% confidence intervals (CIs) calculated via bootstrapping are presented.

Variables	Observations (n)	Raters	Spearman’s rank correlation		Inter-rater reliability (Kappa)			ICC		
			Spearman ρ	p-value	Statistic	z	p	Agreement	Consistency	95% CI (Bootstrap) Lower–Upper
A: TP	180	2	0.531	<0.001	0.589	3.640	<0.001	0.531	0.531	0.232–0.765
B: TO/TC	168	2	0.872	<0.001	0.899	8.600	<0.001	0.871	0.870	0.722–0.970
C: TO/OT	216	2	0.767	<0.001	0.761	6.170	<0.001	0.762	0.764	0.601–0.877
D: TM	120	2	0.721	<0.001	0.720	7.100	<0.001	0.722	0.721	0.579–0.846

Inter-rater reliability

**Figure 3:**
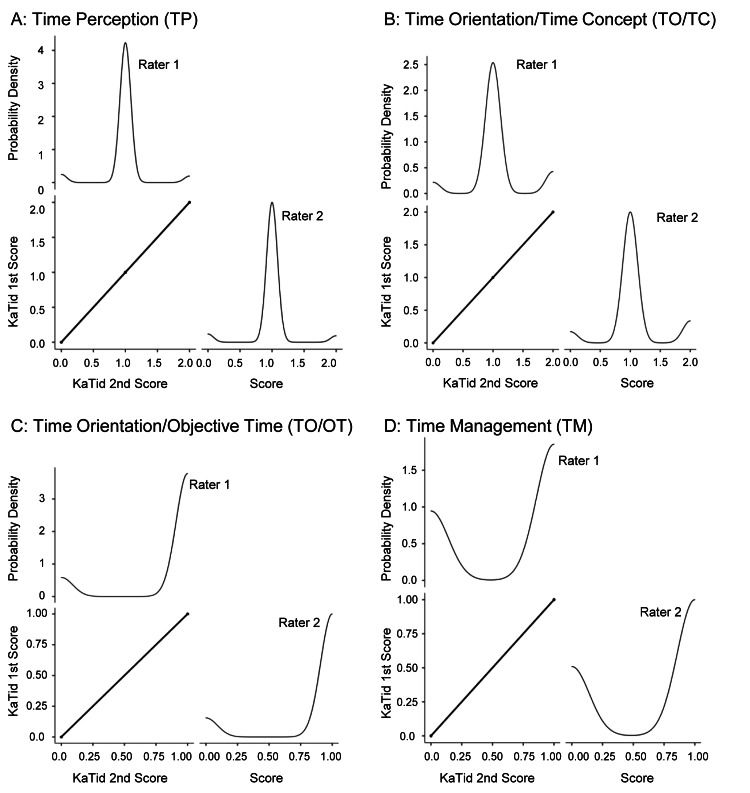
Inter-rater reliability scatterplots and probability density curves These figures illustrate the inter-rater reliability and probability density of the scores (0, 1, 2) for the assessments performed by raters 1 and 2 across the four domains: TP, TO/TC, TO/OT, and TM. Spearman’s rank correlation is illustrated in the scatterplots. Probability density curves represent the score distributions between the two raters.

Inter-rater reliability assessment included 12 participants, with each child rated twice by two raters across four domains (A: 15 items, B: 14, C: 18, D: 10). Bland-Altman plots confirmed that the two raters’ scores were identical for all domains (A: TP, B: TO/TC, C: TO/OT, D: TM), indicating no random or systematic errors. The plots revealed minimal mean differences and narrow limits of agreement, with no observable heteroscedasticity, supporting a high level of consistency between raters. Spearman’s rank correlation coefficient (ρ) was 1.000 for all domains. Figure 3 illustrates the scatter plots and probability density curves for both raters. Table 3 summarizes the validation results, including Spearman’s rank correlation, inter-rater reliability (κ), ICCs, and bootstrap analyses. Kappa and ICC values for inter-rater reliability were 1.00 for all domains. The bootstrap method, performed with 1,000 resamples, yielded 95% confidence intervals ranging from 1.00 to 1.00 for all domains, indicating perfect agreement. These results strongly support the reliability and consistency of scoring across raters and domains.

**Table 3:** Inter-rater reliability verification Note: This table summarizes the inter-rater reliability of the KaTid-Child Japanese version (KaTid-C-J) across four domains: time perception (TP), time orientation/time concept (TO/TC), time orientation/objective time (TO/OT), and time management (TM). Spearman’s rank correlation coefficients (ρ), kappa values, intraclass correlation coefficients (ICCs), and 95% confidence intervals (CIs) calculated via bootstrapping are presented.

Variables	Observations (n)	Raters	Spearman’s rank correlation		Inter-rater reliability (Kappa)			ICC		
			Spearman ρ	p-value	Statistic	z	p	Agreement	Consistency	95% CI (Bootstrap) Lower–Upper
A: TP	360	2	1	<0.001	1.000	8.750	<0.001	1.000	1.000	1.00–1.00
B: TO/TC	336	2	1	<0.001	1.000	13.20	<0.001	1.000	1.000	1.00–1.00
C: TO/OT	432	2	1	<0.001	1.000	11.40	<0.001	1.000	1.000	1.00–1.00
D: TM	240	2	1	<0.001	1.000	13.90	<0.001	1.000	1.000	1.00–1.00

Comparison of KaTid-C-J and KaTid-C

**Figure 4:**
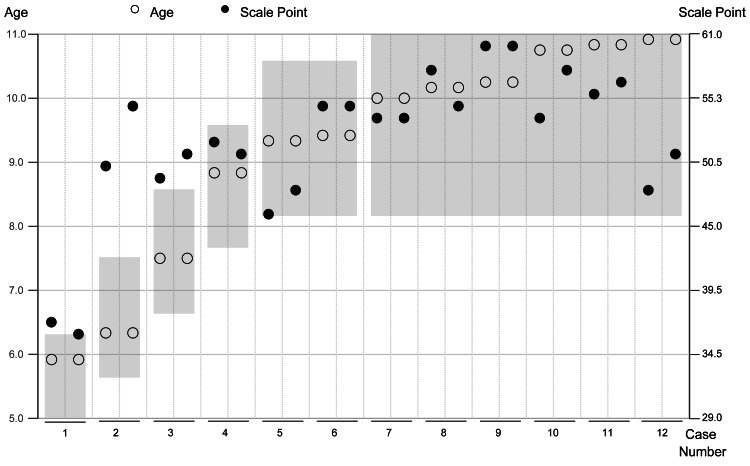
Comparison between the original version and KaTid-C-J White (○) and black dots (●) represent the chronological age at the time of assessment and scale points for each case, respectively. The left and right vertical axes represent the age and scale points, respectively. The horizontal axis corresponds to case numbers 1-12. Gray bands indicate the standard age range as calculated from the original data. KaTid-C-J: Japanese version of the KaTid (Kit for Assessment of Time Processing Ability)-Child

Figure 4 illustrates the assessment results for each case sorted by age. In each case, two measurements obtained on the same day were plotted, and white (○) and black (●) dots indicated the actual age (living age) and ability scores converted to scale points, respectively. Results of the first and second assessments are presented on the left and right sides of the plot, respectively. For example, in Case 1, the second assessment results were lower than the first one. In Case 2, the second assessment result was higher than the first one. The gray band indicated the standard age range, and the plots beyond indicated the ability values that deviated from the standard. For example, five of the plots in Cases 1-3 for ages ages 5-7 years exceeded this band, which indicated higher ability scores relative to their actual ages.

Table 4 summarizes the results of the two assessments of the 12 participants. For each case, the agreement between the original data and actual age was evaluated, which demonstrated the percentage of cases that agreed with the age range of the original data. One assessment result for participants aged five to seven years was consistent with the same age range, and the remaining five results were evaluated as older than the age range of the original data. In addition, all the assessment results of participants aged 8-10 years were compatible with the same age range. Overall, in 12 × 2 measurements, 19 times were compatible with the age band of the original version, and five times were not. In the age group analysis, χ2 test results demonstrated that the overall goodness of fit was 8.17, 2.67, and 1.00 for all ages, 5-7, and 8-10 year age groups, respectively (Table 5).

**Table 4:** Comparison between the KaTid-Child Japanese version (KaTid-C-J) and the original Swedish version Note: This table summarizes the comparison of developmental age bands between the KaTid-Child Japanese version (KaTid-C-J) and the original Swedish version. For each participant, the agreement between actual chronological age and the corresponding ability scale points was evaluated. The table shows the number and percentage of assessments consistent with the age range of the original version across two measurement sessions.

Case	Assessment	Chronological age (in years and months)	Total raw scores	Scale points	Age (Decimal system)	Scale Points (Age)	Age band conformity
1	1	5y 11m	37	54	5.9	6.5	Misfit
	2	5y 11m	36	51	5.9	6.3	Fit
2	1	6y 4m	50	87	6.3	8.9	Misfit
	2	6y 4m	55	93	6.3	9.9	Misfit
3	1	7y 6m	49	85	7.5	8.8	Misfit
	2	7y 6m	51	87	7.5	9.1	Misfit
4	1	8y 10m	52	89	8.8	9.3	Fit
	2	8y 10m	51	87	8.8	9.1	Fit
5	1	9y 4m	46	77	9.3	8.2	Fit
	2	9y 4m	48	82	9.3	8.6	Fit
6	1	9y 5m	55	93	9.4	9.9	Fit
	2	9y 5m	55	93	9.4	9.9	Fit
7	1	10y 0m	54	92	10.0	9.7	Fit
	2	10y 0m	54	92	10.0	9.7	Fit
8	1	10y 2m	58	97	10.2	10.4	Fit
	2	10y 2m	55	93	10.2	9.9	Fit
9	1	10y 3m	60	99	10.3	10.8	Fit
	2	10y 3m	60	99	10.3	10.8	Fit
10	1	10y 9m	54	92	10.8	9.7	Fit
	2	10y 9m	58	97	10.8	10.4	Fit
11	1	10y 10m	56	93	10.8	10.1	Fit
	2	10y 10m	57	94	10.8	10.3	Fit
12	1	10y 11m	48	82	10.9	8.6	Fit
	2	10y 11m	51	87	10.9	9.1	Fit

**Table 5:** Validation of children by age (stratified χ² test) Note: This table presents the results of the stratified chi-squared (χ²) tests comparing the developmental age bands between the KaTid-Child Japanese version (KaTid-C-J) and the original Swedish version. The analyses were performed for the entire sample and separately for the 5-7-year and 8-10-year age groups. The χ² values indicate the goodness of fit between the observed and expected distributions of developmental age consistency.

Variables	Age band conformity	Count	Proportion	χ2 Value	df	p
All ages	Fit	19	0.792	8.17	1	0.004
	Misfit	5	0.208			
5–7 years	Fit	1	0.167	2.67	1	0.102
	Misfit	5	0.833			
8–10 years	Fit	18	1.000	1.00	-	-
	Misfit	0	0.000			

## Discussion

We evaluated the reliability of the KaTid-C-J in assessing Japanese children’s time perception, time orientation, and time management abilities. This study focused on three main aspects: test-retest reliability, inter-rater reliability, and comparison between the original Swedish and Japanese versions.

Test-retest reliability

This study demonstrated high intra-rater reliability for the KaTid-C-J when utilized by a single examiner. Specifically, the TO/TC, TO/OT, and TM domains exhibited substantial agreement. However, the TP domain revealed only moderate agreement. A subtest that required children to stop a stopwatch at exactly 10 seconds without visual cues demonstrated low consistency, as previously observed in both the original Swedish and Japanese versions [11,13].

Inter-rater reliability

The KaTid-C-J demonstrated complete agreement across all the domains when scored by two examiners, which suggested clear and consistent scoring criteria. High inter-rater reliability indicates that trained examiners can use the Japanese version to reliably assess Japanese children. However, such perfect agreement (ICC and kappa values of 1.00) may be partly attributable to the homogeneity of the sample and the limited range of observed scores, rather than reflecting absolute agreement under real-world conditions. This suggests that future studies with larger and more diverse samples may yield more nuanced reliability estimates. Bland-Altman plots further supported the high agreement, but a more detailed interpretation of the mean differences and limits of agreement would provide additional insight into measurement error and potential bias.

Comparison with the original Swedish version

This study sought to elucidate the disparities in outcomes between the Japanese version and the original Swedish version. While the results for children aged 8-10 years were comparable between the two versions, the 5-7-year age group scored higher in the Japanese version. This discrepancy may also be influenced by linguistic and cultural factors. Notably, the Japanese and Swedish languages utilize distinct naming conventions for months, which may also contribute to variations in results. In Japanese, months are expressed using numerical sequences, such as January being ichi-gatsu (“month one”) and February being ni-gatsu (“month two”). In contrast, English, like other Western languages, employs unique, noun-based names for months (e.g., January, February). The direct associative connection that Japanese children establish between these names and numerical order in Japanese might facilitate month-naming tasks at a younger age. This linguistic structure could have contributed to the higher scores observed in the Japanese version for younger children. Other possible contributing factors - such as the small sample size and developmental variability - should also be considered when interpreting these differences.

Study limitations

The study design did not completely adhere to the COnsensus-based Standards for the selection of health Measurement Instruments (COSMIN) risk of bias checklist; several measurement properties (e.g., content, structural, cross-cultural, criterion, and construct validities; internal consistency; responsiveness) were not assessed [18]. The sample size (n = 12) was far below the recommended 100 per group, with a wide age range (5-10 years), uneven gender distribution (10 boys, 2 girls), and recruitment limited to Saitama Prefecture via snowball sampling during the COVID-19 pandemic. These factors limit both internal and external validity.

The retest interval was short (30 minutes to one hour), which may have increased memory effects; future studies should use longer intervals (e.g., 5-10 days). In addition, one rater received direct training from the original author, while the other was trained secondarily, which may have influenced consistency. The perfect inter-rater agreement (ICC/kappa = 1.00) is statistically unusual and could reflect sample homogeneity, limited score variability, or other methodological factors.

Additionally, while the KaTid-C was developed to assess time processing ability across a broad pediatric population, including those with and without developmental disabilities, this pilot study was conducted exclusively with typically developing children. Although this approach allowed for controlled examination of reliability metrics, the findings may not directly generalize to clinical populations with time-processing difficulties. Recent research comparing children with and without ASD has identified specific patterns of time processing difficulties, underscoring the need for tailored support [19]. Future validation studies should include children with various developmental conditions to fully evaluate the applicability and clinical utility of the KaTid-C-J in diverse settings, and stratify by narrower age bands.

Finally, this study did not examine gender differences, although prior work in Japan suggests such differences in developmental milestones (e.g., girls excelling earlier in certain tasks at 5-6 years and again at 7-8 years [20]). Their potential impact on time concept development remains unknown and merits investigation.

Future directions

To generalize the use of the KaTid-C-J, increasing the number of qualified examiners is essential. This can be achieved by creating a comprehensive manual and offering lectures to ensure consistent implementation. In addition, simplifying it for online administration can reduce the burden on participants and increase accessibility. These steps will increase the sample size and facilitate standardization of the KaTid-C-J for wider use in Japan. Furthermore, accurate assessment is a prerequisite for effective intervention. Evidence suggests that structured interventions, such as time-management training programs, can effectively enhance these skills in individuals with cognitive challenges [21].

## Conclusions

This pilot study demonstrated that trained examiners could use the KaTid-C-J to reliably assess Japanese children’s time-related abilities. Both the intra- and inter-rater reliabilities were high, indicating that the KaTid-C-J shows promise as a reliable instrument. However, given the pilot nature of the study, these results should be interpreted with caution. Future research should aim to recruit larger and more demographically diverse samples, stratify participants into narrower age bands, and ensure standardized training for all raters. Such efforts will enhance the tool’s internal and external validity and facilitate robust cross-cultural comparisons with the original Swedish version.
